# Cohort Profile Update: The Norwegian Mother, Father and Child Cohort (MoBa)

**DOI:** 10.1093/ije/dyaf139

**Published:** 2025-08-20

**Authors:** Ragnhild E Brandlistuen, Dana Kristjansson, Elin Alsaker, Ragnhild Valen, Even Birkeland, Ellen C Røyrvik, Christian M Page, Maria Aamelfot, Sille Vangbæk, Helga Ask, Alexandra Havdahl, Anne Lise Brantsæter, Guri Rortveit, Siri E Håberg, Per Magnus

**Affiliations:** The Norwegian Mother, Father, and Child Cohort Study (MoBa), Norwegian Institute of Public Health, Oslo, Norway; Department of Child Health and Development, Norwegian Institute of Public Health, Oslo, Norway; PROMENTA Research Center, Department of Psychology, University of Oslo, Oslo, Norway; Department of Genetics and Bioinformatics, Norwegian Institute of Public Health, Oslo, Norway; Centre for Fertility and Health, Norwegian Institute of Public Health, Oslo, Norway; The Norwegian Mother, Father, and Child Cohort Study (MoBa), Norwegian Institute of Public Health, Oslo, Norway; The Norwegian Mother, Father, and Child Cohort Study (MoBa), Norwegian Institute of Public Health, Oslo, Norway; Department of Genetics and Bioinformatics, Norwegian Institute of Public Health, Oslo, Norway; The Norwegian Mother, Father, and Child Cohort Study (MoBa), Norwegian Institute of Public Health, Oslo, Norway; Department of Genetics and Bioinformatics, Norwegian Institute of Public Health, Oslo, Norway; The Norwegian Mother, Father, and Child Cohort Study (MoBa), Norwegian Institute of Public Health, Oslo, Norway; Department of Genetics and Bioinformatics, Norwegian Institute of Public Health, Oslo, Norway; Department of Clinical Science, University of Bergen, Bergen, Norway; Department of Aging and Physical Health, Norwegian Institute of Public Health, Oslo, Norway; Department of Biobanks, Norwegian Institute of Public Health, Oslo, Norway; The Norwegian Mother, Father, and Child Cohort Study (MoBa), Norwegian Institute of Public Health, Oslo, Norway; PsychGen Center for Genetic Epidemiology and Mental Health, Norwegian Institute of Public Health, Oslo, Norway; Department of Child Health and Development, Norwegian Institute of Public Health, Oslo, Norway; PsychGen Center for Genetic Epidemiology and Mental Health, Norwegian Institute of Public Health, Oslo, Norway; Department of Food Safety, Norwegian Institute of Public Health, Oslo, Norway; Norwegian Institute of Public Health, Oslo, Norway; Centre for Fertility and Health, Norwegian Institute of Public Health, Oslo, Norway; Centre for Fertility and Health, Norwegian Institute of Public Health, Oslo, Norway

**Keywords:** MoBa, longitudinal, cohort, childhood, young adulthood, parents, multigenerational, pregnancy

Key Features

- The Norwegian Mother, Father and Child Cohort Study (MoBa) is a large population-based cohort and biobank for research on health and development.
- MoBa includes data on 94 834 mothers, 75 229 fathers, and 113 632 children (currently aged 15–25 years) recruited during pregnancy between 1999 and 2008, with continuing follow-up from a life-course perspective.
- Up to 25 years of follow-up with linkages to national registries provides unique research opportunities. Recent data collections among children aged 13–25 years and parents aged 32–86 years include questionnaires on health, lifestyle, fertility, COVID-19, cognitive tests, and clinical measurements.
- Genotyped data are available for 225 667 MoBa participants, including ∼30 000 full mother–father–child trios. The biodata resource also offers large-scale datasets (*n* > 10 000) on the epigenome and metabolome and smaller datasets (*n* = 100–10 000) on biomarkers from blood, plasma, urine, and serum.
- MoBa data access is given through helsedata.no; see https://www.fhi.no/en/ch/studies/moba/for-forskere-artikler/research-and-data-access/. New collaborations are welcome.

## The original cohort

The Norwegian Mother, Father and Child Cohort Study (MoBa), initially named the Norwegian Mother and Child Study, was launched in the 1990s by epidemiologists at the Norwegian Institute of Public Health, studying pregnancy outcomes from the Medical Birth Registry of Norway [1]. The primary aim was to identify causes of diseases by collecting comprehensive exposure and outcome data from fetal life onwards [2]. Recruitment began in 1999 with a postal invitation and the first questionnaire was sent prior to the routine ultrasound examination in the 17th week of pregnancy. Biological material was collected during the ultrasound appointment. In 2008, the goal of recruiting 100 000 pregnancies was achieved and recruitment concluded, with the last MoBa baby born in July 2009. Previous MoBa cohort profiles have described the initial sampling and waves of data collection up to 2016 [2, 3].

## What is the reason for the new data collection?

Since 2016, data-collection efforts in MoBa have been initiated to update and enrich existing datasets, providing crucial information on further development and health outcomes, particularly in the offspring generation. Adolescence and early adulthood are life stages characterized by significant social, physical, and emotional changes that can impact lifelong health and wellbeing. The new data collections among MoBa teens (aged 16–17 years) and young adults (aged 18–25 years) encompass a wide range of topics, including physical and mental health, pubertal development, health behaviors, pain, career and family plans, living situations, and social media use. Through a collaboration with the Norwegian University of Science and Technology, MoBa has, for the first time, included a web-based neuropsychological test battery as part of the new data collection to provide reliable measures of cognitive function [4, 5].

The fertility rate has decreased substantially, both in Norway and globally, over the past few decades [6], making the causes and consequences of childlessness important fields of research. Data on reproductive health indicators in young adulthood are being collected through questionnaires and clinical examinations to gain insights into the status of reproductive health in young adults today and into factors influencing fertility, such as hormonal status, environmental exposures, and lifestyle factors.

When the COVID-19 pandemic hit in early 2020, billions of people were affected by the infection and by national and regional strategies to prevent its spread. A biweekly data collection in MoBa was initiated in March 2020 and invitations were sent to all mothers and fathers as well as to 16- to 17-year-olds, to monitor symptoms and to study potential impacts of the pandemic. Additional blood samples were collected in 2020, 2021, and 2023. Important research questions included the effects of vaccination and COVID-19 infection on short- and long-term health, including post-COVID-19 symptoms, as well as mental health trajectories during the pandemic [7].

## What will be the new areas of research?

Longitudinal follow-up of young people aged 15–25 years and their parents (currently at the mean age of 51 years) enables research on a broad range of health conditions for which new knowledge can be of significant societal importance. This includes the development of obesity and type 1 diabetes, rising adolescent mental health issues, increasing frequencies of neurodevelopmental conditions such as autism and attention-deficit/hyperactivity disorder (ADHD), the development of immunological diseases, cardiovascular disease, cancers, post-COVID-19 symptoms, declining fertility rates, digital life and social media use, cognition, education, and labor-market participation. Participant consent allows linkage to high-quality national registries covering a broad range of outcomes.

The unique triad structure (mother, father, and child(ren)) provides the opportunity to study both genetic and environmental aspects of human development. With one of the world’s largest samples of longitudinal family trio data, MoBa offers unique possibilities for advancing research through family-based designs (including twins, siblings, maternal and paternal comparisons/negative control) and genetic designs (including within-family Mendelian randomization (MR) or polygenic MR). These designs can support causal relationships by reducing or eliminating unmeasured and unknown confounding factors due to familial and demographic characteristics [8]. MoBa allows the integration of genetic, multi-omics, environmental, and social factors to understand the early-life antecedents of lifelong health and well-being by using nuanced and comprehensive data and measurements on trios.

In 2025, a new brain-imaging and psychomotor-functioning study will commence in MoBa, aiming to recruit >3000 of the young adult participants to provide detailed and in-depth data on brain functioning. Together with the web-based neuropsychological test battery, brain health and cognition will become increasingly important areas of research in MoBa.

## Who is in the cohort?

MoBa now contains data on 94 834 mothers, 75 220 fathers, and 113 632 adolescents and young adult offspring. The data include 112 304 pregnancies, among which are 1922 sets of twins and 21 sets of triplets. Of the original study participants, 99.3% are still participating in MoBa. Information from various registries can be linked to data from the participants for follow-up; 91% are still active, meaning that they consent to receiving invitations to new data collections.

In 2025, the second-generation participants are between the ages of 15 and 25 years and the parents are between 32 and 86 years old. Table 1 shows the number of participants and response rates for the various data collections. The response rates on questionnaire data collections range from 77% for the first MoBa COVID-19 parent questionnaire to 20% for the ongoing young-adult-years questionnaires. Participants who continue to respond are typically older, female, more highly educated, less likely to smoke, and have lower body mass index [9].

**Table 1.** Overview of recent (2017–25) MoBa data collections.

	Main topics covered	Collection period	Participants (*n*)^a^	Participation (%)
Youth Dietary Questionnaire (food frequency)	Nutrition, diet, physical activity	2017–23	46 429	49
14 years	Mental health and health behavior	2017–23	26 373	29
14 years mothers	Mental health and health behavior	2017–23	29 045	32
MoBaYoung 16–17 years				
Form 1	Mental health, life satisfaction	2019–ongoing	31 881	40
Form 2	Sexual health, harassment, bullying, self-harm	2019–ongoing	19 461	59
Form 3	School engagement, social relations, activities, social media use	2019–ongoing	15 597	48
Form 4	Family socio-economic status, major life events	2019–ongoing	15 280	48
Form 5	Substance use, diet	2019–ongoing	15 924	51
Form 6	Sleep, eating disorders, mental health	2019–ongoing	14 019	46
Form 7	Disorders, treatment	2019–ongoing	13 013	33
Form 8	Self-esteem, psychotic symptoms	2019–ongoing	11 219	38
Body map	Pain	2019–ongoing	11 830	43
Young adult				
18 years	Social functioning, health behavior, mental health	2022–ongoing	14 303	24
19+ years	Education, aspirations, global uncertainty	2023–ongoing	10 631	19
20+ year	Medication use, allergies, disease, cosmetic use, health behavior	2024–ongoing	9313	23
18–25 years	Health, health behavior, relationships, fertility	2024–ongoing	13 091	23
Psychometric tests parents	Complex reaction time, working memory, executive function, general cognitive ability (matrix test)	2023	41 842	27
Psychometric tests adolescents	Complex reaction time, working memory, executive function, general cognitive ability (matrix test)	2021–23	9641	16
COVID-19 parents^b^	Infection, vaccination, side effects	2020–ongoing	114 304 Round 1	77 Round 1
			40 505 Round 58	72 Round 58
COVID-19 offspring^b^	Infection, vaccination, side effects	2020–21	8765 Round 1	51 Round 1
			714 Round 29	42 Round 29
Maternal questionnaire (age 45+ years)	Fertility, lifestyle, menopause	2021–22	25 853	42
Paternal questionnaire (age 45+ years)	Fertility, lifestyle	2021–22	16 284	29
Maternal health	Fertility, lifestyle, cosmetic surgery, tattoos	2024	42 371	50
Paternal health	Fertility, lifestyle, cosmetic surgery, tattoos	2024	23 061	35

a All numbers are based on the status in 2025.

b The COVID-19 data collections were collected biweekly or monthly throughout the pandemic for the parental generation and during the first year for the offspring generation aged 16–18 years. The sample was adjusted to include only participants who had answered previous rounds.

## What has been measured?

### Questionnaires, neuropsychological tests, samples, and clinical examinations

Since the previous cohort profile update in 2016, the MoBaYoung (16–17), Young adult (18–25), MoBaCovid19, MoBa Brain Health, and several other questionnaires have covered a broad range of topics and measures (Table 1). Mental health and subjective wellbeing have been measured by using the Hopkins Symptom Checklist (SCL5 and SCL10) [10], Generalized Anxiety Disorder scale-7 (GAD-7) [11], and Cantril self-anchoring scale (Cantril Ladder) [12]. In addition to the questionnaire measures, complex reaction time, working memory, executive function, and general cognitive ability (matrix test) were measured by using the neuropsychological test battery “Memoro [13].”

In a sub-study with young adults in MoBa, additional blood and urine samples were collected along with other physiological measurements including blood pressure, an electrocardiograph, and an ultrasound for ovaries and testicles for a clinical study on fertility and health, initiated in 2022. Participants between the ages of 18 and 25 years have been invited to take part in examinations at three fertility clinics around the country. The study is ongoing and aims to recruit 2500 participants. So far, 651 women and 83 men have been examined.

Table 5 gives an overview of all available biospecimen samples collected from parental and offspring participants at different time points.

**Table 5.** Overview of biological material in MoBa.

Participant	Material	Count (*n*)
Pregnant mother at ultrasound (week 17th–20th) (K11 and/or K12^a^)	Whole blood	97 017
	Whole blood^a^	96 822
	Plasma EDTA	77 595
	Plasma EDTA^a^	98 417
	DNA	69 608
	Urine^a^	79 071
Mother 0–3 days after birth	Whole blood	80 470
	Plasma EDTA	85 489
	DNA	86 446
Child at birth (umbilical cord)	Whole blood	89 296
	Plasma EDTA	50 778
	DNA	91 210
	RNA Tempus	45 259
Father at ultrasound	Whole blood	71 289
	Plasma EDTA	72 072
	DNA	72 527
Child 5–10 years	Primary teeth^b^	26 728
Child 3 years (Autism Birth Cohort Study)	DNA	870
	Whole blood	681
	Plasma	881
Child 8 years (Language8-Study)	DNA	640
Child 7–10 years (BraPust)	Plasma (EDTA)	447
	Serum	451
	DNA	450
Mother 2016 Environmental Biobank	Whole blood	657
	Red blood cells	657
	Plasma	657
	Serum	655
	Urine	660
	RNA	577
Father 2016 Environmental Biobank	Whole blood	496
	Red blood cells	497
	Plasma	497
	Serum	497
	Urine	500
	RNA	426
Child 2016 Environmental Biobank	Whole blood	654
	Red blood cells	655
	Plasma	604
	Serum	658
	Urine	668
	RNA	576
Mother 2024–2025 Environmental Biobank	Whole blood	1075
	Red blood cells	1060
	Plasma	1077
	Serum	1077
	Urine	1075
Father 2024–2025 Environmental Biobank	Whole blood	574
	Red blood cells	565
	Plasma	574
	Serum	574
	Urine	571
Child 2024–2025 Environmental Biobank	Whole blood	512
	Red blood cells	508
	Plasma	516
	Serum	520
	Urine	517
Mother 2020 (COVID-19)	DNA	3546
	Plasma	2641
Father 2020 (COVID-19)	DNA	2873
	Plasma	2096
Child 2020 (COVID-19)	DNA	2866
	Plasma	2059
Mother 2021	DNA	743
	Plasma	742
Father 2021	DNA	627
	Plasma	627
Child 2021	DNA	767
	Plasma	764
Mother, post COVID-19 2023	DNA	163
	Plasma	163
Father, post COVID-19 2023	DNA	108
	Plasma	108
Female child, 18–27 years old, 2022–ongoing	DNA	561
	Whole blood	567
	Plasma	478
	Urine	508
	RNA	576
Male child, 18–27 years old, 2024–ongoing	DNA	10
	Whole blood	17
	Plasma	17
	Urine	10
	RNA	17
	Semen	16

a K11 (K1 no. 1) was the original collection. K12 (K1 no. 2) was funded by the National Institute of Environmental Health Sciences.

b Primary teeth are stored in the MoBaTooth Biobank in Bergen.

EDTA, ethylenediaminetetraacetic acid; RNA, ribonucleic acid.

### The Biological Data Resource in MoBa

Multiple categories of biological data on MoBa participants continue to be generated from research projects in MoBa. The bio-datasets have been produced by using various analysis technologies applied to whole-blood, urine, plasma, and deoxyribonucleic acid (DNA) samples. Researchers and MoBa data managers prepare genetic, epigenetic, protein, and metabolomic data for reuse in line with the findable, accessible, interoperable, and reusable (FAIR) principles. Tables 2–4 give an overview of existing biological data currently available. A complete description of all currently available MoBa biodata and biomarkers can be found at the Biological Data Resource in MoBa—NIPH [14].

**Table 2.** Genomics data in MoBa.

Data type	Participant type(s)	Tissue type	Official name	*n* ^a^ ^,b^
Genotyping data	Mothers, fathers, children	DNA (whole blood and cord blood)	snp001–snp019	225 667
DNA methylation	Mothers, fathers, children	DNA (whole blood and cord blood)	met001–met013	17 449
Polymorphisms related to one-carbon, folate, or homocysteine metabolism	Mothers	DNA (whole blood)	Gtp001	2907
Polymorphisms in genes related to xenobiotic metabolism	Mothers, children	DNA (whole blood)	Gtp002	2011
Polymorphisms related to one-carbon, folate, or homocysteine metabolism	Mothers, fathers, children	DNA (whole blood)	Gtp003	1424
*KIR* and fetal *HLA-C* genes	Mothers, children	DNA (whole blood)	Gtp004	596
*KIR* and fetal *HLA-C* genes	Mothers, children	DNA (whole blood)	Gtp005	1949
Mitochondrial DNA	Children	DNA (whole blood)	Mit001	861
Leukocyte telomere length	Mothers, fathers, children	DNA (whole blood)	Tel001	4788
Zygosity	Children (twins)	DNA (cord blood)	Zyg001	618

a All numbers are based on the status in 2025.

b Subject to withdrawn consent.

**Table 3.** Key parameters for proteomics data available in MoBa.

Biomarker type(s)	Participant type(s)	Tissue type	Case selection criteria	Control criteria	Analytic method(s)	Official name	*N*
Hormone, metabolic, and inflammatory	Mothers	Plasma	Subfecundity	Random	Olympus AU400e Clinical Chemistry Analyzer; immunoassay	Pro001	947
Dietary, renal, and inflammatory	Mothers	Plasma	Preeclampsia	Random	Olympus AU400e chemistry immuno-analyzer	Pro002	1137
Inflammatory, nutritional, metabolic, and thyroid	Mothers	Plasma	Random	n/a	ARCHITECT^®^ 8200ci integrated analyser	Pro003	2971
Glycated hemoglobin	Mothers	Whole blood	Random	n/a	ARCHITECT^®^ ci8200 System	Pro004	2964
Immunology	Mothers, children	Plasma	ADHD	Random	Immunoassay	Pro005	2257
Thyroid function	Mothers	Plasma	ADHD	Random	Quantitative chemiluminescent immunoassay	Pro006	1133
IgM and IgG against cytomegalovirus	Mothers	Plasma	Preeclampsia	Healthy pregnant women	ELISA	Pro007	2451
Sex hormones, thyroid, cardiometabolic	Children	Serum	Random		ARCHITECT^®^ 8200ci integrated analyser	Pro008	630
Dietary and thyroid biomarkers	Children	Plasma	Random		ARCHITECT^®^ ci8200	Pro009	659
Adiponectin and leptin	Children	Plasma	Random		ELISA	Pro010	659
Ferrin and soluble transferrin receptor	Children	Plasma	Diabetes, type I		ELISA	Pro011	1118

ELISA, enzyme-linked immunosorbent assay.

**Table 4.** Metabolomic biomarkers in MoBa.

Metabolite type(s)	Tissue type	Case selection criteria	Control criteria	Analytic method(s)	Name	***N*** ^a^ ^,b^
Metabolic biomarkers	Plasma	ART		NMR	Mab002	15 234
Vitamin B, one-carbon metabolites	Plasma	Random	n/a	GC–MS, LC–MS, and microbiological assay	Mab001	3000
Vitamins A, D2, D3, and E	Plasma	Random	n/a	LC–MS	Mab004	2434
Vitamins A, D2, D3, and E	Plasma	Language delay at 3 years old		LC–MS	Mab018	527
Folate and fatty acids	Plasma	Language delay at 3 years old		GC–MS, LC–MS, and microbiological assay	Mab019	531
Metabolic disease biomarkers	Plasma	Renal cell carcinoma	Cancer-free	LC–ESI–MS/MS and FIA–ESI–MS/MS	Mab006	161
Per- and polyfluoroalkyl substances (PFAS)	Plasma	Subfecundity	Time to pregnancy <12 months	LC–MS	Mab003	947
PFAS	Plasma	ADHD	Random	LC–MS/MS	Mab008	2324
PFAS	Plasma	Cerebral palsy, autism spectrum disorder, epilepsy	Random	LC–MS/MS	Mab010	1104
Perfluorinated compounds	Plasma	Preeclampsia	Random	LC–MS	Mab005	1137
Heavy metals and essential elements	Whole blood	ADHD	Random	ICP–SFMS	Mab007	1878
Heavy metals and essential elements	Whole blood	Random	n/a	ICP–MS, CVAFS	Mab011	2984
Heavy metals and essential elements	Whole blood	Cerebral palsy, autism spectrum disorder, epilepsy	Random	ICP–SFMS	Mab009	1050
Nutritional, stress, renal, and metabolic disease markers	Urine	Random	n/a	ARCHITECT^®^ 8200ci integrated analyser	Mab012	2981
Carotenoids	Plasma	Random	n/a	HPLC	Mab013	2975
Iodine	Urine	Random	n/a	ICP–MS	Mab014	2981
Organophosphate esters	Urine	ADHD	Random	UPLC	Mab015	1143
Organophosphate esters	Urine	ADHD	Random	UPLC	Mab016	1145
Phthalates	Urine	ADHD	Random	LC–MS/MS	Mab017	1149

a Subject to withdrawn consent.

b Unique individuals.

ART, assisted reproductive technology; PFAS, per- and polyfluoroalkyl substances; NMR, nuclear magnetic resonance; GC–MS, gas chromotography-mass spectrometry; LC–MS, liquid chromatography-mass spectrometry; LC-ESI-MS/MS, liquid chromatography electrospray ionization tandem mass spectrometry; FIA-ESI-MS/MS, flow injection analysis-tandem mass spectrometry; ICP-SFMS, inductively coupled plasma–sector field mass spectrometry; HPLC, high-performance liquid chromatography; UPLC, ultra-high performance liquid chromatography; CVAFS, cold vapor atomic fluorescence spectrophotometry.

Currently, genotype data on 225 667 individuals, including parents and children, are available. DNA methylation at birth has been generated for >17 000 children from multiple studies examining prenatal exposures. Polymorphisms related to one-carbon, folate, homocysteine, or xenobiotic metabolism have also been measured. Telomere length—a biomarker often associated with aging—has been measured in 4788 mothers, fathers, and children (∼1600 trios) (Table 2).

Zygosity determination for all twin births in MoBa has been conducted through genotyping (Table 2) and questionnaires. Additionally, studies have measured metabolomic (Table 4) and proteomic (Table 3) biomarkers, including cortisol levels and thyroid hormones, vitamins (A, D, and E), per- and polyfluoroalkyl substances (PFAS), heavy metals, and essential elements (e.g. lead, mercury, arsenic, zinc, and iodine).

## What has it found? Key findings and publications

MoBa data are used in multiple research disciplines, from social science to biomedical and toxicological science. Over 1200 scientific publications using MoBa data, covering a wide range of topics, have been published so far. A complete overview can be found at the MoBa publications website [15].

Some recent highlights include:

- Variants in the leptin receptor and leptin genes have transient and dynamic effects on body mass index in infancy and early childhood (0–8 years), suggesting the importance of these genes in healthy infant growth. These findings also suggest that weight-management intervention should be tailored to the developmental stage and genetic profile of patients [16].
- Blood samples from MoBa mothers were used to examine maternal concentrations of PFAS—a group of highly persistent man-made chemicals. Results from MoBa have contributed to the regulation and ban of some of these chemicals by documenting bioaccumulation in women in the years after pregnancy and lactation [17], and showed that increased exposure to PFAS was associated with lower vaccine response in children [18].
- A common missense variant in the *UGT1A4* gene significantly reduces the risk of jaundice in newborns. This variant affects bilirubin conjugation through regulation of the UGT1A1 enzyme in the intestines, highlighting distinct genetic mechanisms for bilirubin clearance in neonates compared with adults [19].
- Newborns conceived through assisted reproductive technology exhibit widespread differences in DNA methylation compared with naturally conceived newborns. These differences are found in genes related to growth, neurodevelopment, and other health outcomes, and could not be explained by parental subfertility [20].
- Assortative mating on traits such as educational attainment, height, and depression results in genetic similarities between partners and extended family members, including siblings-in-law and co-siblings-in-law [21].
- Long-term prenatal exposure to paracetamol is associated with significant DNA methylation differences in children diagnosed with ADHD, with affected genes and pathways linked to ADHD, neural development, neurotransmission, oxidative stress, and neurological processes [22].
- Pregnant women in Norway have low iodine intake and insufficient iodine status. Low iodine intake is associated with adverse pregnancy outcomes [23] and compromised neurocognitive development up to 8 years of age [24, 25].
- Different early-childhood risk profiles (0–3 years) are longitudinally associated with later mental health and academic outcomes (8–14 years). Kindergarten factors exert protective effects [26].
- There has been a general increase in symptoms of eating disorders among 14- to 16-year-olds over time. Adjusting for this trend, the COVID-19 pandemic seems to have exacerbated eating problems, particularly among girls [27].
- Stricter public health measures and quarantine during the COVID-19 pandemic were associated with adolescent mental distress [28].
- Booster vaccination before Omicron infection substantially reduced both neurocognitive and cardiorespiratory symptoms occurring ≥3 months after Omicron infection [29].

## What are the main strengths and weaknesses?

The MoBa cohort study boasts several unique features and strengths. Firstly, its large scale and longitudinal design, encompassing >113 000 children and their parents with ongoing follow-up (currently 15–25 years), provides robust statistical power to study multiple outcomes and exposures, including rare conditions such as neurodevelopmental disorders, cancers, and genetic disorders. The phenotypically rich data collected allow detailed and nuanced analyses of various traits and their associations, while the genotype data enable comprehensive genetic analyses to explore contributing genetic factors and gene–environment interactions. The 25-year longitudinal follow-up from pregnancy onwards offers invaluable insights into changes and effects over time. Additionally, the multigenerational aspect of the study provides an opportunity to investigate hereditary and familial health patterns.

A major strength is the ability to link cohort participants to individual-level high-quality health registries and national records, providing objective outcome measures for diagnoses, prescriptions, hospitalizations, as well as education, work participation, and more. This linkage enables comprehensive follow-up of health outcomes, even for the participants who do not complete all questionnaire responses. The family- and population-based genetic designs support a variety of methodological approaches, such as sibling control design, trio genome-wide complex trait analysis, MR, polygenic MR, and multivariable MR and mediation, which are instrumental in disentangling causal factors.

However, MoBa has some limitations. The initial parental inclusion rate of 41% provides the potential for nonresponse bias and may limit the generalizability of the findings. However, associations are not necessarily biased [30]. Self-reported data from questionnaires can suffer from recall bias and inaccuracies. Health registries provide objective measures on all participants, but they are limited to people using the healthcare services. Still, they are likely to have high coverage, as there is universal public healthcare for all citizens in Norway and nearly all healthcare is recorded in the registries. Clinical test data are not available for all MoBa participants—only for participants who have taken part in different sub-studies. The complexity and variety of methodological designs necessary to disentangle causal factors could still leave residual confounding, complicating the establishment of definitive causation.

## Can I get hold of the data? Where can I find out more?

Researchers in Norway can apply for access at the Norwegian health data sources webpage (helsedata.no). Researchers not affiliated with a Norwegian research institution may collaborate with, and apply through, a Norwegian researcher. MoBa aims to provide researchers worldwide with access to FAIR data and samples for research on health and development. Instrument documentation is provided on the NIPH website [31]. All use of data and biological material from MoBa is subject to Norwegian law. More information on how to apply for access to data and biological material from MoBa can be found on the MoBa website at the Norwegian Institute of Public Health [32]. For new collaborative projects, please contact Ragnhild Brandlistuen at Ragnhild.Brandlistuen@fhi.no.

## Ethics approval

The establishment of MoBa and initial data collection were based on a license from the Norwegian Data Protection Agency and approval from the Regional Committees for Medical and Health Research Ethics. The MoBa cohort is currently regulated by the Norwegian Health Registry Act. Each new research project needs to obtain ethical approval for using the data collected in MoBa for the specific research questions of interest.

## Data Availability

Data from the Norwegian Mother, Father and Child Cohort Study is managed by the Norwegian Institute of Public Health. Access requires approval from the Regional Committees for Medical and Health Research Ethics (REC), compliance with GDPR, and data owner approval. Participant consent does not allow individual-level data storage in repositories or journals. Researchers seeking access for replication must apply via www.helsedata.no.
